# Efficient Detection of Occlusion prior to Robust Face Recognition

**DOI:** 10.1155/2014/519158

**Published:** 2014-01-16

**Authors:** Rui Min, Abdenour Hadid, Jean-Luc Dugelay

**Affiliations:** ^1^Department of Multimedia Communications, EURECOM, 450 Route des Chappes, 06410 Biot, France; ^2^Center for Machine Vision Research, Department of Computer Science and Engineering, University of Oulu, P.O. Box 4500, 90014 Oulu, Finland

## Abstract

While there has been an enormous amount of research on face recognition under pose/illumination/expression changes and image degradations, problems caused by occlusions attracted relatively less attention. Facial occlusions, due, for example, to sunglasses, hat/cap, scarf, and beard, can significantly deteriorate performances of face recognition systems in uncontrolled environments such as video surveillance. The goal of this paper is to explore face recognition in the presence of partial occlusions, with emphasis on real-world scenarios (e.g., sunglasses and scarf). In this paper, we propose an efficient approach which consists of first analysing the presence of potential occlusion on a face and then conducting face recognition on the nonoccluded facial regions based on selective local Gabor binary patterns. Experiments demonstrate that the proposed method outperforms the state-of-the-art works including KLD-LGBPHS, S-LNMF, OA-LBP, and RSC. Furthermore, performances of the proposed approach are evaluated under illumination and extreme facial expression changes provide also significant results.

## 1. Introduction

Face recognition [1], the least intrusive biometric technique in terms of acquisition, has been applied to a wide range of commercial and law enforcement applications. State-of-the-art face recognition systems perform with high accuracy under controlled environments, but performances drastically decrease in practical conditions such as video surveillance of crowded environments or large camera networks. The main problems are due to changes in facial expressions, illumination conditions, face pose variations, and presence of occlusions. With emphasis on real-world scenarios, in the last decade, problems related to pose/illumination/expression changes and image degradations have been widely investigated in the literature. In contrast, problems caused by occlusions received relatively less investigations, although facial occlusion is quite common in real-world applications especially when individuals are not cooperative with the system such as in video surveillance applications.

**Figure 1:**
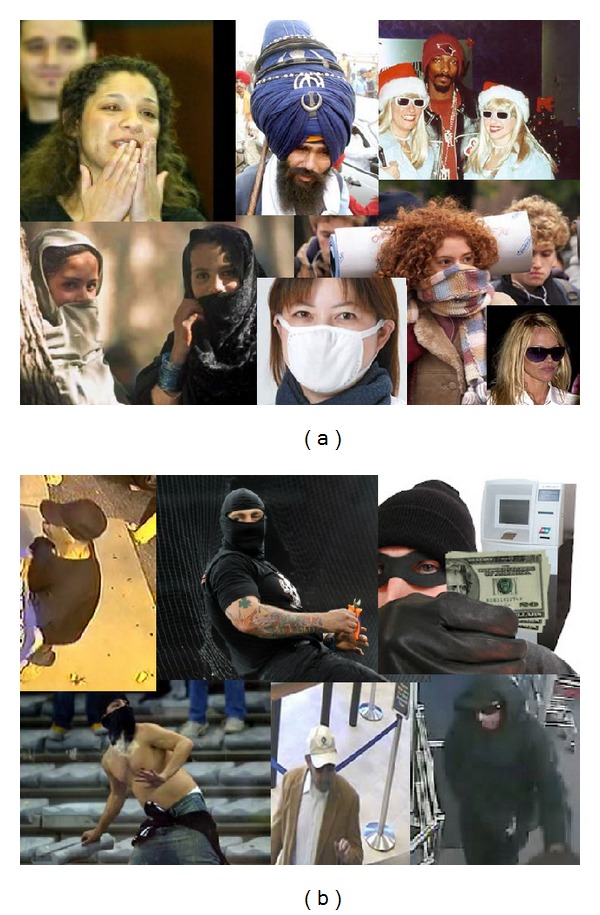
Illustration of different types of facial occlusions: (a) ordinary facial occlusions in daily life; (b) facial occlusions related to severe security issues (ATM crimes, football hooligans, etc.).

Facial occlusions may occur for several intentional or undeliberate reasons (see Figure 1). For example, facial accessories like sunglasses, scarf, facial make-up, and hat/cap are quite common in daily life. Medical mask, hard hat, and helmet are required in many restricted environments (e.g., hospital and construction areas). Some other people do wear veils for religious convictions or cultural habits. In addition, facial occlusions are often related to several severe security issues. Football hooligans and ATM criminals tend to wear scarves and/or sunglasses to prevent their faces from being recognized. Bank robbers and shop thieves usually wear a cap when entering places where they commit illegal actions.

Because partial occlusions can greatly change the original appearance of a face image, it can significantly deteriorate performances of classical face recognition systems (such as [2–4], since the face representations are thus largely distorted). To control partial occlusion is a critical issue to achieve robust face recognition. Most of the literature works [5–17] focus on finding corruption-tolerant features or classifiers to reduce the effect of partial occlusions in face representation. However, information from the occluded parts can still hinder the recognition performance. Recently, researchers [18–21] demonstrated that prior knowledge about the occlusion (e.g., type, location, and size) can be used to exclude the information from occluded parts, so as to greatly improve the recognition rate. Hence, explicit occlusion analysis is an important step in occlusion-robust face recognition. In this paper, we propose an occlusion analysis method to improve local Gabor binary pattern based face recognition [11], which outperforms literature works including [17–21].

The proposed approach consists of first detecting and segmenting occluded parts (e.g., sunglasses/scarves) and then applying face recognition on the nonoccluded facial regions. To do so, the presence of occlusion is first analysed in the patch-level using Gabor wavelets, PCA and SVM. Then we segment the occluded part more precisely from the other facial regions by a generalized Potts model Markov random field (GPM-MRF) [22]. This allows us to identify the presence of occlusion at the pixel-level so as to preserve as much as possible face information for the recognition. After the computation of an occlusion mask indicating which pixel in a face image is occluded, we propose a variant of local Gabor binary pattern histogram sequences (LGBPHS) [11] to efficiently represent occluded faces by excluding features extracted from the occluded pixels. Finally, we compared our approach with traditional approaches [2, 4, 11], our previous results [21], and state-of-the-art methods [13, 19, 20] on AR face database [23] and obtained the best results. Our experiments also suggested that, in comparison with weighting based method [20], occlusion exclusion (i.e., weighting as 0 or 1) is more appropriate to handle the occlusion problem in face recognition.

The rest of this paper is structured as follows. First, we review the related works in Section 2. Then, the proposed approach is described in Section 3.  Section 4 presents the experimental results and analysis. Finally, we draw the conclusion and discuss future directions in Section 5.

## 2. Related Works

The traditional methodology to address face recognition under occlusion is to find corruption-tolerant features or classifiers. Toward this goal, numerous previous works confirmed that locally emphasized algorithms are less sensitive to partial occlusions. Penev and Atick [5] proposed the local feature analysis (LFA) to extract local features by second order statistics. Martínez [6] proposed a probabilistic approach (AMM) which can compensate for partially occluded faces. Tan et al. [7] extended Martinez's work by using the self-organizing map (SOM) to learn the subspace instead of using the mixture of Gaussians. In [8], Kim et al. proposed a method named locally salient ICA (LS-ICA) which only employs locally salient information in constructing ICA basis. In [9], Fidler et al. presented a method which combines the reconstructive and discriminative models by constructing a basis containing the complete discriminative information. Park et al. [10] proposed to use a line feature based face attributed relational graph (ARG) model which encodes the whole geometric structure information and local features of a face. Zhang et al. [11] proposed a nonstatistical face representation—local gabor binary pattern histogram sequence (LGBPHS), to exploit the multiresolution and multiorientation Gabor decomposition. In [12], Jia and Martinez proposed the use of partial support vector machines (PSVM) in scenarios where occlusions may occur in both the training and testing sets.

More recently, facial occlusion handling under the sparse representation based classification (SRC) [13] framework has demonstrated impressive performances in face recognition with occlusions. The idea of using SRC for occluded face recognition is first introduced by Wright et al. [13], where an occluded face is represented as a linear combination of the whole face gallery added by a vector of errors (occlusion) in the pixel-level and the classification is achieved by L1 minimization. Zhou et al. [14] extend [13] by including a Markov Random Fields (MRF) model to enforce spatial continuity for the additive error vector to address contiguous occlusions. In [15], Yang and Zhang applied compressible image Gabor features instead of original image pixels as the feature vector used in SRC to reduce computations in the presence of occlusions. Liao and Jain [16] incorporated the SIFT descriptor into the SRC framework to achieve alignment free identification. Yang et al. [17] proposed a robust sparse coding (RSC) method which seeks the maximum likelihood estimation (MLE) solution of the sparse coding problem for non-Gaussian/Laplacian occlusions in an iterative manner. Even though the SRC based methods achieve significant identification results on occluded faces from standard face databases (i.e., AR face database [23]), the prerequisite of those methods relies on the large number of training samples of each identity with sufficient variations. But in many practical face recognition scenarios, the training samples of each subject are often insufficient (the “curse of the dimensionality” [24] problem, in the extreme case, only one template face per subject is available).

Lately, a few works have revealed that prior knowledge of occlusions can significantly improve the accuracy of local feature/local component based face recognition. Rama et al. [18] empirically showed that prior knowledge about occlusion (manually annotated) can improve Eigenface in local patches. In [19], Oh et al. have proposed an algorithm based on local nonnegative matrix factorization (LNMF) [25], named selective LNMF (S-LNMF) that automatically detects the presence of occlusion in local patches; face matching is then performed by selecting LNMF representation in the nonoccluded patches. Zhang et al. [20] proposed to use Kullback-Leibler divergence (KLD) to estimate the probability distribution of occlusions in the feature space, so as to improve the standard LGBPHS based method [11] for partially occluded face. In our preliminary study [21], we also demonstrated that explicit occlusion analysis can greatly improve LBP based face recognition. In these studies, [18, 19, 21] discard all information from the components which are occluded, whereas [20] assigns a weight (between 0 and 1) to each component. In this paper, we consider the first case as occlusion exclusion and the later one as occlusion weighting (note that occlusion exclusion can be regarded as a special case of occlusion weighting, where the weights are either 0 or 1). Because many of the algorithms we have discussed so far will be extensively analysed and compared in the experiments section, we summarize and categorize the literature works in Table 1 (for which abbreviations will be used in later sections).

Based on our preliminary work [21], in this paper, we propose a complete and fully automatic framework to improve face recognition in the presence of partial occlusions. Besides the occlusion detection module (which was introduced in [21]) which can detect the presence of occlusion in patch-level, we adopted GPM-MRF to detect occlusion in pixel-level to facilitate later recognition. We then propose a customized corruption-tolerant local descriptor selective LGBPHS which summarizes features from nonoccluded pixels for efficient face representation and recognition. Unlike [11, 20], our approach applies occlusion exclusion (by assigning weights as 0 or 1) based on our explicit occlusion analysis. Our results demonstrate that occlusion exclusion is more efficient than occlusion weighting, since weighting based methods still preserve some information from the occluded region. In addition, because the proposed occlusion analysis is an independent module from the face matching part and no model learning step (such as Eigenface [2], Fisherface [3] or SRC [13]) is required in our approach, the proposed method is not limited by the number of training samples. As a consequence, unlike SRC based methods [13–17], the proposed approach can be applied to face recognition with very limited training samples (one sample per person in the extreme case).

## 3. Approach

**Figure 2:**
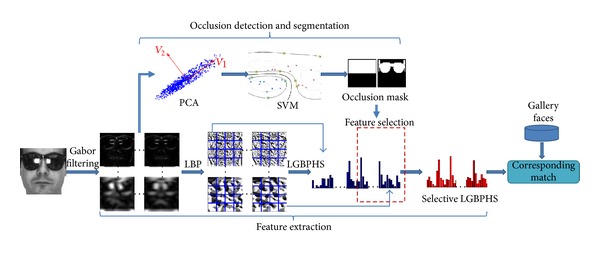
System flowchart.

A comprehensive overview of the proposed system is given in Figure 2. Given a target (i.e., probe) face image (which can be occluded or not) to be recognized, the possible presence of occlusion is first analysed. The probe image is divided into a number of facial components for occlusion detection. Each component is individually analysed by an occlusion detection module. As a result, potential occluded facial components are identified. Then, an occlusion mask is generated by a more precise segmentation approach to supervise the feature extraction and matching process. Based on the resulting occlusion mask, its LGBPHS representation is computed using the features extracted from the nonoccluded region only, namely, selective LGBPHS. The recognition is performed by comparing the selective LGBPHS from the probe image against selective LGBPHS from the template images using the same occlusion mask. The nearest neighbour (NN) classifier and Chi-square (*χ* ^2^) distance are adopted for the recognition.

### 3.1. Occlusion Detection in Local Patches

**Figure 3:**
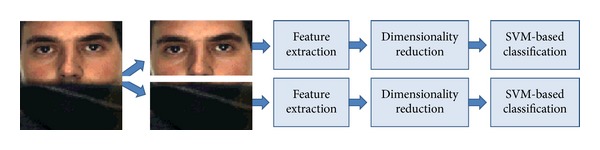
Our occlusion detection scheme.

As depicted in Figure 3, our occlusion detection starts by dividing the face image into different facial components. The number and the shape of the components are determined by the nature of the occlusion. Since our focus in this work is scarf and sunglasses, we accordingly divide the face images into two equal components as shown in Figure 3. The upper part is used for analysing the presence of sunglasses while the lower part is used for detecting scarf.

#### 3.1.1. Gabor Wavelet Based Feature Extraction

Gabor wavelets are used for extracting features from the potentially occluded regions. The choice of using Gabor wavelets is motivated by their biological relevance, discriminative power, and computational properties. A Gabor wavelet consists of a complex sinusoidal carrier and a Gaussian envelope which can be written as

(1) $$\begin{array}{c} \psi_{\mu ,\gamma }\left(z\right)=\frac{{||k_{\mu ,\gamma }||}^{2}}{\delta^{2}}e^{\left(-{||k_{\mu ,\gamma }||}^{2}{||z||}^{2}/2\delta^{2}\right)}\left[e^{ik_{\mu ,\gamma }z}-e^{-\delta^{2}/2}\right], \end{array}$$

where *μ* and *γ* are the orientation and scale of the Gabor kernels, *z* = (*P*, *Q*) is the size of the kernel window, ||·|| denotes the norm operator, *k*_*μ*,*γ*_ = *k*_*γ*_ *e* ^*iϕ*_*μ*_^ is a wave vector, where *k*_*γ*_ = *k*_max_/*f* ^*γ*^ and *ϕ*_*μ*_ = *πμ*/8, *k*_max_ is the maximum frequency, and *f* is the spacing factor between kernels in the frequency domain.

In our system, we set *z* = (20,20), *δ* = 2*π*, *k*_max_ = *π*/2, and $f=\sqrt{2}$ as also suggested in [20]. Five scales *γ* ∈ [0,…, 4] and eight orientations *μ* ∈ [0,…, 7] are selected to extract the Gabor features. In total, 40 Gabor wavelets are generated.

Once the Gabor wavelets are generated, feature extraction is performed by convolving the wavelets with the face image *I*:

(2) $$\begin{array}{c} C_{\mu ,\gamma }\left(x,y\right)=I\left(x,y\right)*\psi_{\mu ,\gamma }\left(z\right). \end{array}$$

Because the phase information of this transform is time varying, we only explore the magnitude information. The computed Gabor magnitude pictures (GMPs) thus form a set *Ω* = {*C*_*μ*,*γ*_, *μ* ∈ [0,7], *γ* ∈ [0,4]}, in which an augmented feature vector is constructed by concatenating all the GMPs. The obtained feature vector is downsampled by a factor *λ* (here *λ* = 5) for further processing. Note that GMPs are not only used in occlusion detection but also used to compute the face representation selective LGBPHS as described in Section 3.3.

#### 3.1.2. Dimensionality Reduction Using PCA

Because the size of extracted Gabor feature is rather big, in order to reduce the dimension of the feature vectors while preserving its discriminative power, we apply principal component analysis (PCA) to maximize the variance in the projected subspace for the Gabor features. To compute the PCA subspace, we consider a training dataset consisting of feature vectors from both occluded and nonoccluded image patches. Let us denote the feature vectors from the nonoccluded patches by *X* ^*c*^ and let us denote the feature vectors from the occluded patches by *X* ^*s*^. The training dataset *S* can be formed as: *S* = {*X*_1_^*c*^, *X*_2_^*c*^,…, *X*_*M*/2_^*c*^, *X*_*M*/2+1_^*s*^,…, *X*_*M*_^2^}, where *M* is the size of the training dataset. The eigenvectors associated with the *k* largest eigenvalues of $(S-\overset{-}{S}){\left(S-\overset{-}{S}\right)}^{T}$ (the covariance matrix of *S*) are thus computed to describe the eigenspace. The Gabor wavelet based features are then projected onto the computed eigenspace for dimensionality reduction.

#### 3.1.3. SVM Based Occlusion Detection

Occlusion detection can be cast as a two-class classification problem. Since nonlinear support vector machines (SVM) are proven to be a powerful tool for discriminating 2 classes of high dimensional data, we adopted then a nonlinear SVM classifier for occlusion detection. Let us consider a training set consisting of *N* pairs {*x*_*i*_, *y*_*i*_}_*i*=1_^*N*^, where *x*_*i*_ refers to a reduced feature vector of a facial component *i*, and *y*_*i*_ ∈ {−1,1} is the label which indicates if the sample *x*_*i*_ is occluded or not. SVM finds the optimal separating hyper-plane {*α*_*i*_, *i* ∈ [1, *N*]} by solving a quadratic programming problem [26] and predicts the label of an unknown face *x* by

(3) $$\begin{array}{c} f\left(x\right)=\mathrm{sign}\left(\overset{N}{\underset{j=1}{\sum }}\alpha_{j}y_{j}K\left(x_{j},x\right)+b\right), \end{array}$$

where {*x*_*j*_, *j* ∈ [1, *N*]} are the support vectors. Nonlinear SVM applies kernels *K*(*x*_*i*_, *x*_*j*_) to fit the maximum-margin hyper-plane in a transformed feature space. In our system, the Radial Basis Function (RBF) kernel is used. The implementation of the nonlinear SVM is provided by LIBSVM [27].

### 3.2. Occlusion Segmentation

In order to efficiently exploit the information of facial occlusion for face recognition, we generate a binary mask *β* (1 for occluded pixels and 0 for nonoccluded pixels) indicating the location of occluded pixels to facilitate later feature extraction and matching in the recognition phase. This mask generation process is called occlusion segmentation. To generate an accurate occlusion mask (which can remove the occluded part meanwhile preserving as much as information from the nonoccluded part), we adopt a generalized Potts model Markov random field (GPM-MRF) [22] to enforce structural information (shape) of occlusion, so as to identify if a given pixel is occluded or not.

Our occlusion segmentation can be formulated as a typical energy-minimization problem in computer vision. Let us consider the face image (consists of multiple facial patches) as an undirected adjacency graph *G* = (*V*, *E*) where *V* = {*v*_*i*_, *i* ∈ [1, *N*]} denotes the set of *N* pixels (vertex) and *E* denotes the edges between neighbouring pixels. Given a set of observations *O* = {*o*_1_,…, *o*_*N*_} corresponding to the set of vertex *V*, we want to assign a label (occluded: 1, nonoccluded: −1) to each vertex. We model the set of labels *L* = {*l*_*i*_, *i* ∈ [1, *N*]} (discrete random variables taking values in Λ = {−1,1}) as a first-order Markov random field. The structural prior is incorporated into the MRF by a generalized Potts model. Then our goal is to find the label set $\hat{L}$ that maximizes the posterior probability *P*(*L* | *O*), which can be achieved by the maximum a posteriori (MAP) estimation [28] that maximizes the joint probability *P*(*O*, *L*), where

(4) P(O,L)=P(O ∣ L)P(L)=1Zexp(−U(L)/T),

where *Z* is the partition function and *T* is the temperature. *U*(*L*) is the sum of potentials from all cliques *C* = {*c*_*i*_, *i* ∈ [1, *N*]}, which can be written as

(5) U(L)=∑ci∈CVc(L)=∑li∈LΨ(oi ∣ li)+ω∑(li,lj)∈EΦ(li,lj),

where *ω* is a weighting parameter controlling the importance of MRF prior (the choice of *ω* is based on experiments on a validation set). The unary potential Ψ is defined by the likelihood function:

(6) $$\begin{array}{c} \Psi \left(o_{i}\;|\;l_{i}\right)=-\ln p\left(o_{i}\;|\;l_{i}\right). \end{array}$$

We approximate the occlusion likelihood (*l* = 1) as follows:

(7) $$\begin{array}{c} p\left(o\;|\;l=1\right)=\{\begin{array}{cc} {\text{e}}^{-1} & \text{if}\;o\;>\;\tau \\ 1-{\text{e}}^{-1} & \text{else}, \end{array} \end{array}$$

where 0 < *τ* < 1 and the face likelihood (*l* = −1) as a constant *c* ∈ [0,1]:

(8) $$\begin{array}{c} p\left(o\;|\;l=-1\right)=c. \end{array}$$

**Figure 4:**
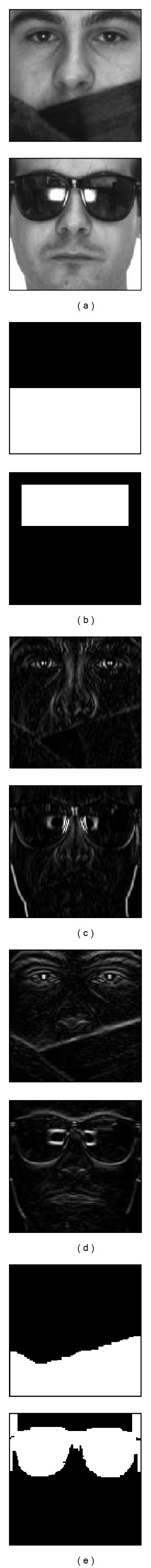
Illustration of our occlusion segmentation: (a) examples of faces occluded by scarf and sunglasses; (b) initial guess of the observation set according to the results from our occlusion detector; ((c)(d)) the visualization of *u*(*i*, *j*) in horizontal and vertical directions, respectively; (e) the generated occlusion masks (*ω* = 150).

Because we have already identified the type of occlusion (obtained by our occlusion detector), we can give an initial guess of observations *O* (the seed of occlusion mask, see Figure 4(b)) to each type of occlusions. The structural information is enforced into this initial guess via the isotropic MRF prior *P*(*L*), where the pairwise potential Φ(*l*_*i*_, *l*_*j*_) has the form of generalized Potts model as defined in [22]:

(9) $$\begin{array}{c} \Phi \left(l_{i},l_{j}\right)=u\left(i,j\right)\cdot \left(1-\xi \left(l_{i}-l_{j}\right)\right), \end{array}$$

where *ξ*(·) represents the unit impulse function; then, Φ(*l*_*i*_, *l*_*j*_) = 2*u*(*i*, *j*) if *i* and *j* have different labels (*l*_*i*_ ≠ *l*_*j*_) and zero otherwise. The structural information *u*(*i*, *j*) is obtained as the first-order derivative after Gaussian filtering (with kernel size (5,5)) from the original image. Note that maximizing the joint probability *P*(*O*, *L*) is equivalent to minimizing the cliques potential *U*(*L*), and this energy minimization problem can be solved exactly using graph cuts [29–31] in polynomial time. The obtained label set $\hat{L}$ (see Figure 4(e)) is converted to the segmentation mask *β* ∈ [0,1] for later recognition task.

### 3.3. Selective LGBPHS Based Face Representation and Recognition

To perform the recognition step, we propose a variant of LGBPHS [11] based face representation (namely, selective LGBPHS) which selects features from nonoccluded pixels only. The choice of using LGBPHS based representation is based on the following facts: (1) it takes the advantage of both Gabor decomposition (multiresolution and multiorientation for enhanced discriminative power) [6] and LBP description (robustness to monotonic gray scale changes caused by, e.g., illumination variations) [4]; (2) block-based histogram representation makes it robust to face misalignment and pose variations to some extent; (3) it provides state-of-the-art results in representing and recognizing face patterns under occluded conditions [11, 20]; (4) Gabor features in LGBPHS share the same computation as in our occlusion detection module.

#### 3.3.1. LGBPHS

Given a face image and its Gabor magnitudes pictures (GMPs) *Ω* = {*C*_*μ*,*γ*_, *μ* ∈ [0,7], *γ* ∈ [0,4]} computed by the method described in Section 3.1.2, the GMPs are further encoded by an LBP operator, resulting in a new feature description—local Gabor binary patterns (LGBP). The LBP operator forms labels for the image pixels by thresholding the 3 × 3 neighbourhood of each pixel with the center value and considering the result as a binary number. The histogram of these 2^8^ = 256 different labels can then be used as a texture descriptor. Each bin (LBP code) can be regarded as a microtexton. Local primitives which are codified by these bins include different types of curved edges, spots, and flat areas.

The calculation of LGBP codes is computed in a single scan through each GMP using the LBP operator. The value of the LGBP code of a pixel at position (*x*_*c*_, *y*_*c*_) of each scale *μ* and orientation *γ* of GMPs is given by

(10) $$\begin{array}{c} \text{LGB}{\text{P}}_{P,R}^{\mu ,\gamma }=\overset{P-1}{\underset{p=0}{\sum }}s\left(g_{p}^{\mu ,\gamma }-g_{c}^{\mu ,\gamma }\right)2^{P}, \end{array}$$

where *g*_*c*_^*μ*,*γ*^ corresponds to the intensity of the center pixel (*x*_*c*_, *y*_*c*_) in the GMP *C*_*μ*,*γ*_, *g*_*p*_^*μ*,*γ*^ refers to the intensities of *P* equally spaced pixels on a circle of radius *R*, and *s* defines a thresholding function as follows:

(11) $$\begin{array}{c} s\left(x\right)=\{\begin{array}{cc} 1 & \text{if}\;x\geq 0\\ 0 & \text{otherwise}. \end{array} \end{array}$$

*μ* × *γ* LGBP maps {*G*_*μ*,*γ*_ *μ* ∈ [0,7], *γ* ∈ [0,4]} are thus generated via the above procedure. In order to exploit the spatial information, each LGBP map *G*_*μ*,*γ*_ is first divided into *r* local regions from which histograms are extracted and concatenated into an enhanced histogram *h*_*μ*,*γ*_ = (*h*_*μ*,*γ*,1_,…, *h*_*μ*,*γ*,*r*_). Then the LGBPHS is obtained by concatenating all enhanced histograms *H* = (*h*_0,0_,…, *h*_4,7_).

#### 3.3.2. Selective LGBPHS

The original LGBPHS summarizes the information from all pixels of a face image. Given an occlusion mask *β* (generated by our occlusion segmentation), our interest is to extract features from the nonoccluded pixels only. Hence, we compute each bin *h*_*i*_ of the histogram representation using a masking strategy as follows:

(12) $$\begin{array}{c} h_{i}=\underset{x,y}{\sum }\left(1-\beta \left(x,y\right)\right)\cdot I\left\{f\left(x,y\right)=i\right\},\;\forall i\in \left[0,2^{P}-1\right], \end{array}$$

where *i* is the *i*th LGBP code, *h*_*i*_ is the number of nonoccluded pixels with code *i*, and

(13) $$\begin{array}{c} I\left\{A\right\}=\{\begin{array}{cc} 1 & A\;\text{is}\;\text{true}\\ 0 & A\;\text{is}\;\text{false}. \end{array} \end{array}$$

Then the histograms extracted from all local regions of all GMPs are concatenated into the final representation, which is named selective LGBPHS. During matching, selective LGBPHS is computed for both probe face and template faces, based on the occlusion mask generated from the probe.

In the selective LGBPHS description, a face is represented in four different levels of locality: the LBP labels for the histogram contain information about the patterns on a pixel-level; the labels are summed over a small region to produce information on a regional-level; the regional histograms are concatenated to build a description of each GMP; finally histogram from all GMPs are concatenated to build a global description of the face. This locality property, in addition to the information selective capability, is behind the robustness (to facial occlusions) of the proposed descriptor.

## 4. Experimental Results and Analysis

To evaluate the proposed approach, we performed a set of experiments on AR face database [23] and compared our result against those of seven different methods including Eigenface [2], LBP [4], OA-LBP [21], LGBPHS [11], KLD-LGBPHS [20], S-LNMF [19], and RSC [17]. Among the selected methods, KLD-LGBPHS, S-LNMF, and OA-LBP (our previous work) are the state-of-the-art works which explicitly exploit automatic occlusion analysis (whereas Part-PCA [18] is based on manual annotation) to improve face recognition according to our survey in Section 2. LBP and LGBPHS are selected to represent the locally emphasized methods without explicit occlusion analysis. Because RSC reports the most recent and very competitive result among all SRC based methods [17], we select it as the representative algorithm of SRC based methods for comparison.

### 4.1. Experimental Data and Setup

**Figure 5:**
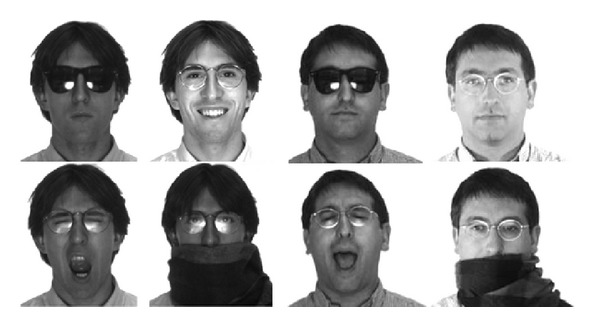
Example of images from the AR face database.

For our experimental analysis, we considered the AR face database [23] which contains a large number of well-organized real-world occlusions. The AR database is the standard testing set for the research of occluded face recognition, and it is used in almost all literature works [6–21]. It contains more than 4000 face images of 126 subjects (70 men and 56 women) with different facial expressions, illumination conditions, and occlusions (sunglasses and scarf). Images were taken under controlled conditions but no restrictions on wearing (clothes, glasses, etc.), make-up, hair style, and so forth were imposed to participants. Each subject participated in two sessions, separated by two weeks (14 days) of time. The original image resolution is 768 × 576 pixels. Some examples of face images from the AR face database are shown in Figure 5. Using eye and nose coordinates, we cropped, normalized, and downsampled the original images into 128 × 128 pixels.

For occlusion detection, we randomly selected 150 nonoccluded faces, 150 faces occluded with scarf, and 150 faces wearing sunglasses for training the PCA space and SVM. The upper parts of the faces with sunglasses are used to train the SVM-based sunglass detector while the lower parts of the faces with scarf are used to train the SVM-based scarf detector. The 150 nonoccluded faces are used in the training of both classifiers.

**Figure 6:**
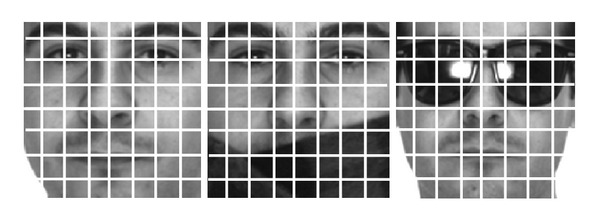
The face images are divided into 64 blocks for selective LGBPHS representation.

For face recognition, the face images are then divided into 64 blocks as shown in Figure 6. The size of each block is 16 × 16 pixels. The selective LGBPHS is extracted using the operator LBP_8,2_^*u*2^ (using only uniform patterns, 8 equally spaced pixels on a circle of radius 2) on the 40 GMPs, yielding feature histograms of 151040 bins.

To test the proposed algorithm, we first selected 240 nonoccluded faces from session 1 of the AR database as the templates images. These nonoccluded faces correspond to 80 subjects (40 males and 40 females), with 3 images per subject under neutral expression, smile, and anger. To build the evaluation set, we considered the corresponding 240 nonoccluded faces from session 2, the 240 faces with sunglasses of session 1, and the 240 faces with scarf of session 1, under three different illuminations conditions.

### 4.2. Results of Occlusion Detection

The proposed occlusion segmentation, feature extraction, and subsequent recognition all rely on the correct occlusion detection. To justify the proposed occlusion detection method, we show the detection rates on all 720 testing images.  Table 2 illustrates the results as a confusion matrix. Note that only 2 images (faces with very bushy beard) from the nonoccluded faces are wrongly classified as faces with scarf. The correctness of our occlusion detection ensures the correct feature selection in the later recognition steps.

### 4.3. Results of Occluded Face Recognition

**Figure 7:**
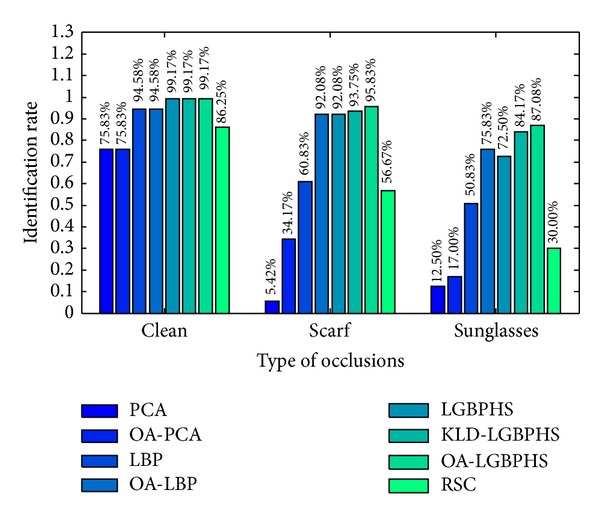
Results of PCA, OA-PCA, LBP, OA-LBP, LGBPHS, KLD-LGBPHS, OA-LGBPHS, and RSC on three different testing sets (faces are clean and faces are occluded by scarf and sunglasses).

Figure 7 shows the face recognition performance of our approach on three different test sets: clean (nonoccluded) faces, faces occluded with scarf, and faces occluded with sunglasses. For comparison, we also report results of the state-of-the-art algorithms (for the name abbreviations, please refer to Table 1) for both standard face recognition and occluded face recognition. Eigenfaces [2] (i.e., PCA) and LBP [4] are among the most popular algorithms for standard face recognition. We also tested the approaches which incorporate our occlusion analysis (OA) with the standard Eigenface and LBP, namely, OA-PCA and OA-LBP [21]. Similarly, we denote the proposed approach by occlusion analysis assisted LGBPHS (OA-LGBPHS). In order to justify that the proposed method is more appropriated for occluded faces, we also tested the standard LGBPHS [11] and its variant KLD-LGBPHS [20] on the same data set, where LGBPHS, KLD-LGBPHS, and OA-LGBPHS apply different preprocessing methods to the same face representation. The method RSC [17] is selected to represent the family of algorithms based on sparse representation [13–17], in which RSC is one of the most robust algorithms according to the reported results. It should be noticed that, in the pool of selected algorithms, KLD-LGBPHS, OA-LBP, and RSC stand for the state-of-the-art algorithms for occluded face recognition in each of the 3 categories as we reviewed in Section 2 (see Table 1).

In Figure 7, it is clear that the proposed approach (OA-LGBPHS) obtains the highest identification rates in all 3 cases (99.17%, 95.83%, and 87.08% for clean, scarf, and sunglass faces, resp.). Without explicit occlusion analysis, facial occlusions such as scarf and sunglasses can greatly deteriorate the recognition results of PCA and LBP; in contrast, OA-PCA and OA-LBP surpass their original algorithms significantly. With a long length feature vector (151040 bins), LGBPHS demonstrates satisfactory robustness to facial occlusions. Without occlusion analysis, LGBPHS can already yield close results to OA-LBP under the occlusion conditions. KLD-LGBPHS improves LGBPHS by associating a weight with each block (which indicates the level of occlusion) to ameliorate the impact from occluded regions and the weight is measured as a deviation of the target block from the pre-defined mean model based on Kullback-Leibler divergence. Although KLD-LGBPHS greatly increases the results in comparison to LGBPHS (especially for faces occluded by sunglasses), its performance is still inferior to OA-LGBPHS. This result reveals that occlusion exclusion is more efficient than occlusion weighting, since distortions due to facial occlusions do not affect the process of recognition when the occluded regions are completely discarded.

Sparse representation based classification (SRC) is well known for its robustness to partial distortions (e.g., noise, occlusion, etc.) as well as its discriminative power. However, it also suffers from the “curse of dimensionality” problem, where in many practical cases, the number of templates (of each identity) is insufficient to support the recovery of correct sparse coefficients. On the given data set (240 training faces, with 3 templates for each identity), robust sparse coding (RSC) yields relatively low identification rates (86.25%, 56.67%, and 30%).

Comparing the results on the test sets of faces with sunglasses and scarves, we notice that most methods (except for PCA) are more sensitive to sunglasses than to scarf. This is an interesting phenomenon which is in agreement with the psychophysical findings indicating that the eyes/eyebrows region plays the most important role in face recognition [32].

### 4.4. Robustness to Other Facial Variations

We compared our proposed approach against OA-LBP and S-LNMF [19] using similar protocol under the more challenging scenario in which the gallery face images are taken from session 1 of AR database while the test sets are taken from session 2. Note that the two sessions were taken at time interval of 14 days. The comparative results of our approach against OA-LBP and S-LNMF are illustrated in Table 3.

The results in Table 3 clearly show that our proposed approach outperforms OA-LBP and S-LNMF in all configurations showing robustness against sunglasses, scarves, screaming, and illumination changes. The robustness of our approach to illumination changes and drastic facial expression is brought by the use of local Gabor binary patterns, while the occlusion detection module significantly enhances the recognition of faces occluded by sunglasses and scarves even with time elapsing.

Please note that we did not provide the comparative results of our approach to all the literature works (according to our survey in Section 2). Instead, we compare our approach to a number of carefully selected methods. Because our method exploits explicit occlusion analysis, KLD-LGBPHS, S-LNMF, and OA-LBP which belong to the same category (see Table 1) are selected for the comparisons in our experiment. RSC is selected to represent the family of SRC based face recognition. Even though LGBPHS is chosen to stand for the locally emphasized algorithms without explicit occlusion analysis, our approach could be directly extended to other local feature/classifier based methods for potential improvements.

## 5. Conclusions

We addressed the problem of face recognition under occlusions caused by scarves and sunglasses. Our proposed approach consisted of first conducting explicit occlusion analysis and then performing face recognition from the nonoccluded regions. The salient contributions of our present work are as follows: (i) a novel framework for improving the recognition of occluded faces is proposed; (ii) state-of-the-art in face recognition under occlusion is reviewed; (iii) a new approach to detect and segment occlusion is thoroughly described; (iv) extensive experimental analysis is conducted, demonstrating significant performance enhancement using the proposed approach compared to the state-of-the-art methods under various configurations including robustness against sunglasses, scarves, nonoccluded faces, screaming, and illumination changes. Although we focused on occlusions caused by sunglasses and scarves, our methodology can be directly extended to other sources of occlusion such as hats, beards, and long hairs. As a future work, it is of interest to extend our approach to address face recognition under general occlusions, including not only the most common ones like sunglasses and scarves but also beards, long hairs, caps, and extreme facial make-ups. Automatic face detection under severe occlusion, such as in video surveillance applications, is also far from being a solved problem and thus deserves thorough investigations.

## Figures and Tables

**Table 1:** Summary of literature works in occluded face recognition.

Category	Abbreviation	Full name/brief description
Locality emphasized features/classifiers.	LFA [5]	Local feature analysis.
	AMM [6]	Gaussian mixture modelling of part-based Eigenface.
	SOM-AMM [7]	Self-organizing map modelling of part-based Eigenface.
	LS-ICA [8]	Local salient-independent component analysis.
	RD-Subspace [9]	Combining reconstructive and discriminative subspace.
	ARG [10]	Attributed relational graph.
	LGBPHS [11]	Local Gabor binary patterns histogram sequence.
	PSVM [12]	Partial support vector machines.

SRC based methods.	SRC [13]	Sparse representation based classification.
	MRF-SRC [14]	Markov random field to enforce spatial continuity in SRC.
	Gabor-SRC [15]	Compressible Gabor feature used in SRC.
	SIFT-SRC [16]	SIFT feature used in SRC.
	RSC [17]	Robust sparse coding.

Explicit occlusion analysis facilitated local feature/local component based methods.	Part-PCA [18]	Occlusion analysis + part-based Eigenface.
	S-LNMF [19]	Selective local nonnegative matrix factorization.
	KLD-LGBPHS [20]	Local Gabor binary patterns based on Kullback-Leibler divergence.
	OA-LBP [21]	Occlusion analysis + LBP (our preliminary work).

**Table 2:** Results of occlusion detection.

	No-occlusion	Scarf	Sunglass	Detection rate
Non occlusion	**238 **	2	0	99.17%
Scarf	0	**240 **	0	100%
Sunglass	0	0	**240 **	100%

**Table 3:** Robustness to different facial variations.

	Sunglasses	Scarf	Scream	Right light
S-LNMF	49%	55%	27%	51%
OA-LBP	54.17%	81.25%	52.50%	86.25%
OA-LGBPHS	**75% **	**92.08% **	**57.50% **	**96.25% **
